# Applying multilevel analysis and the Driver Behavior Questionnaire (DBQ) on unsafe actions under a road safety policy

**DOI:** 10.1371/journal.pone.0277750

**Published:** 2022-11-16

**Authors:** Sajjakaj Jomnonkwao, Savalee Uttra, Buratin Khampirat, Vatanavongs Ratanavaraha

**Affiliations:** 1 School of Transportation Engineering, Institute of Engineering, Suranaree University of Technology, Nakhon Ratchasima, Thailand; 2 School of General Education, Institute of Social Technology, Suranaree University of Technology, Nakhon Ratchasima, Thailand; Tsinghua University, CHINA

## Abstract

The aims of this research are: to investigate and develop a multilevel analysis of unsafe actions or risky behaviors; to study the influence of road safety policy factors on risky behaviors; and to analyze personal characteristics that influence risky behaviors. Data were collected using 1,474 samples from locations countrywide at the district level, including 76 clusters, via the Driver Behavior Questionnaire (DBQ) and road safety policy. The results indicate that, for the district-level model, the participation factor directly and negatively influenced risky behaviors, and government support indirectly had a negative impact through participation. Thus, people’s participation in the area caused a decrease in unsafe behaviors. Meanwhile, safety policy support in the area partially caused people to participate at a significant level. At the personal level, income, having a driver’s license, past violations, and past accidents significantly affected risky behaviors, especially having a driver’s license, which had a negative influence. This meant that people who had a driver’s license facilitated a positive effect in terms of decreasing risky behaviors, while people with past violations and past accidents influenced this situation positively. The more traffic law violations and accidents the participants had, the more they engaged in unsafe actions. Based on the findings, acknowledging and solving the problem of unsafe driving at a spatial level can address the issue by supporting different measures to help people in the area improve the situation. In addition, we should assist people who have a driver’s license by offering them useful training to decrease traffic law violations and inform them about accidents.

## 1. Introduction

### 1.1 Background

Road safety management has been widely mentioned in research. Findings have been applied for road safety policies and improving strategies. Abroad, road safety policy indicates that law enforcement can be enhanced for road safety; for instance, reducing speeding by 10.54%, as speeding is illegal and occurs among 42.8% of drivers in Iran [1], or in relation to drunk driving in Greece [2]. Appropriate and intensive law enforcement leads drivers to obey the law, eventually decreasing the number of road accidents by 50% [3]. Furthermore, unsafe driver behavior has been mentioned in road safety policy.

For example, Stanojević et al. [4] asserted that if law enforcement officers become negligent, there will be an increase in unsafe driving behaviors such as speeding, failure to fasten seatbelts, drunk driving, aggressive behavior, traffic law violations, and many more risky behaviors. Yannis et al. [5] studied law enforcement at the national and regional levels in relation to accidents, focusing on drunk driving. They found that law enforcement was significantly affected. Nevertheless, several studies have implied that traffic enforcement has no effects on accidents and driving behavior [4, 6].

Vehicles, the environment, and road users are well-known as the major factors of accidents, and are important factors for improving road safety problems [7, 8]. Without communities’ cooperation in solving road safety problems by using law enforcement and engineering [9], essential strategies to develop road safety policies cannot be developed [10].

### 1.2 Road safety in Thailand

In 2019, 99,087 accidents occurred in Thailand [11]; based on insurance claims, data from Thailand Statistics of Traffic Accidents at the Provincial Level found that the number of vehicle accident victims totaled 375,564 at the province, district, and sub-district levels. The numbers vary by province (Bangkok: 51,020; Chiang Mai: 16,919; Nakhon Ratchasima: 13,730; Phuket: 8,528) [12]. While considering area conditions, the population size and number of vehicles also differ. However, all areas share a common road safety policy, a central policy enforced nationwide by law enforcement authorities, as well as campaign support from the government to control and solve the problem of road accidents.

The Thailand Road Safety Master Plan (2018–2021) was issued by the Department of Disaster Prevention and Mitigation [13] according to Thailand’s road safety context and provides a bottom-up approach to ensure road safety management leverage at the local level. The structure of road safety management can be classified at the national, provincial, district, and local levels, respectively. Besides, there are some support organizations that encourage road safety activities, covering both the public and private sectors.

As part of the road safety policy operation organized at the 13^th^ Road Safety Seminar, “Invest in Sustainable Road Safety, Brainstorming, Prevention, and Decreasing Road Accidents,” the Thai government announced seven measures to decrease the death rate, and to support the creation of road safety operation centers at the local, provincial, and district levels (Department of Local Administration, speed limits in urban areas, mandatory alcohol consumption tests, and standards for issuing driver’s licenses for youths who ride large motorbikes, with the aim of creating a decade of safer roads [the so-called “Decade of Road Safety Operation, Years 2011–2020”] to decrease the rate of accidents by half by 2020. The government has continuously emphasized prevention to reduce road accidents, particularly by having four prohibitions and two mandatory measures, respectively, as follows: no fast driving, no drunk driving, no driving while sleepy, no talking on the phone while driving, always using a seatbelt while driving, and wearing a helmet while riding a motorbike.

### 1.3 Literature review, analytic framework, and hypothesis

#### 1.3.1 Human factors

Olson and Dewar [7] examined the human factors behind accidents to understand human behavior. First, we have to understand people’s characteristics regarding work, skills, and various qualifications. In terms of road accidents, the six relevant factors identified include basic control, general driving, traffic conditions, roadway characteristics, environment, and vehicle type. Olson and Dewar [7] has mentioned considering human factors, drivers’ perceptions and responses are relevant, such as where they are looking and for how long, different kinds of personal data, emotions, pressure, aggression [14–20], motivation [21], driving skills, risky behaviors [20, 22], social variables [22, 23], driver attitudes [24, 25], gender differences [14, 26, 27], driving experience, tiredness, alcohol consumption [28, 29], drunk driving behavior [30], age differences (i.e., teen, adult, or senior) [14, 27, 31, 32], and other physical characteristics also constitute human factors that can lead to accidents. Accidents also happen due to co-factors between the driver and the vehicle, or humans and the environment, such as roads, and so forth [7].

#### 1.3.2 Unsafe actions

Road and vehicle factors create unsafe conditions and generate the risk of accidents, and human factors are relevant to risky behavior and safety violations [33]. Human factors consist of physical conditions, intelligence, mind, and attitudes, especially driving behaviors, thoughts, and mental states, which cause unsafe actions [34].

Unsafe driver behavior was studied in China [21], and involved an exploration of factors that influenced online car-hailing drivers based on the theory of planned behavior (TPB). Unsafe driver behavior or risky behavior includes errors lapses and violations [35], as seen in the Driver Behavior Questionnaire (DBQ) [36].

Recently, studies on driver behavior have mostly focused on traffic safety. The DBQ, created by Reason et al. [36], has been used to study risky behaviors related to driving, and it has been widely applied in places such as in Denmark [14]; France [15]; the UK [16]; Australia [17]; Serbia [19]; Bulgaria, Romania, and Serbia [37]; and New Zealand [38]. Researchers have found that driving behaviors can be classified into four categories: violations, lapses, errors, and aggressiveness. The modified DBQ, which consists of 25 questions with the four factors of violations, errors, lapses, and aggressiveness, was used to evaluate driving behavior in Thailand [39]; it will be applied in further research for this study.

In addition, the characteristics of risky behaviours include gender-affected bicycle riding behavior [20, 26, 27, 31, 32], differences in attitudes and the motivation for riding or driving [21, 25], age [26, 27, 31, 40], driving skills [41], and having a driver’s license [42–45]. Moreover, past violations [4, 46] and accidents are relevant.

#### 1.3.3 Multilevel analysis

Multilevel structural equation modeling (SEM) entails a combination of SEM and multilevel analysis to study the relationships among the variables relevant to the structural model; their measurement levels are equal to or greater than 2 levels. This technique originated from the work of Muthén [47], who used all levels of the variables studied to combine them and analyze them in one model, meaning that there is no need to analyze two separate processes, as was the case with the former method. The model consists of two sub-models: (1) the between-group model, which involves the causative relationships among macro-level variables or group-level such as policy, school, province, district, and teacher; and (2) the within-group model, which concerns the causative relationships among individual-level variables or micro-level variables such as gender, age, income, and occupation. Then, both sub-models are analyzed together in a multilevel fashion by creating a special latent variable as an average variable at the micro level, as there is a decomposition of the variation of the variables in the multilevel analysis of the study for both the between-group and within-group levels [48]

Several scholars have used the multilevel SEM technique, such as Mohammad and Hadikusumo [49], who studied safety at work to improve workplace behavior. They found that this approach can be used to provide guidance regarding safety management in construction work (at the management level). In addition, this technique helps to improve migrant workers’ capacity for safe behavior (at the technique and human levels). Moreover, Jones and Jørgensen [50] found that age, gender, type of vehicle, road section, alcohol, and time of day affected fatality risk. Okoye et al. [51] studied the Nigerian construction industry and discovered that safety interventions improved programmers’ effectiveness and helped to prevent construction site accidents. Ratanavaraha et al. [48] investigated sightseeing buses by using the school and teacher levels, and determined that service quality positively affected satisfaction, which in turn positively impacted loyalty in a statistically significant way at both the individual and school levels.

Chen and Mu [52] applied multilevel analysis to study injury severity from riding motorcycles among Taiwan’s elders; the regional and individual levels were designed in this study. The findings were effectively used to develop road safety policies or strategies at the local level. As well as Thailand, supposing that multilevel analysis would apply to road safety studies at the district or provincial level, the results might be used as a guideline to develop policies or strategies.

### 1.4 Research objectives, contributions, analytical framework, and hypotheses

This research design is based on the assumption of road accidents caused by humans and the current accident situation in Thailand; related organizations still provide policies or regulations to solve this problem continuously. In other countries, detailed road safety policy was studied. It consists of determining the law, law enforcement by authorities, which affected positive actions or behavior. In addition, law enforcement was implemented continuously and intensively, including projects’ public relations and other regulations. Studying the Thailand road safety master plan illustrated that policies were developed from the centers and distributed to each administrative area (i.e., regions, provinces, districts, sub-districts, and communities), which can lead to conclusions that environmental factors relevant to accidents occurred.

However, human factors were indicated in previous research as causes of accidents such as gender, age, behavior, characteristics, socioeconomic status, having a driver’s license, traffic violations, and accident experiences, which influence human behavior. Hence, human factors will be underlined as a main point in this study. Under a controlled environment called road safety policy, this study does not concentrate on identifying guidelines to develop new road safety policy, but rather aims to examine the influence of road safety policy factors on unsafe actions in order to realize the effect of government policies.

**Fig 1:**
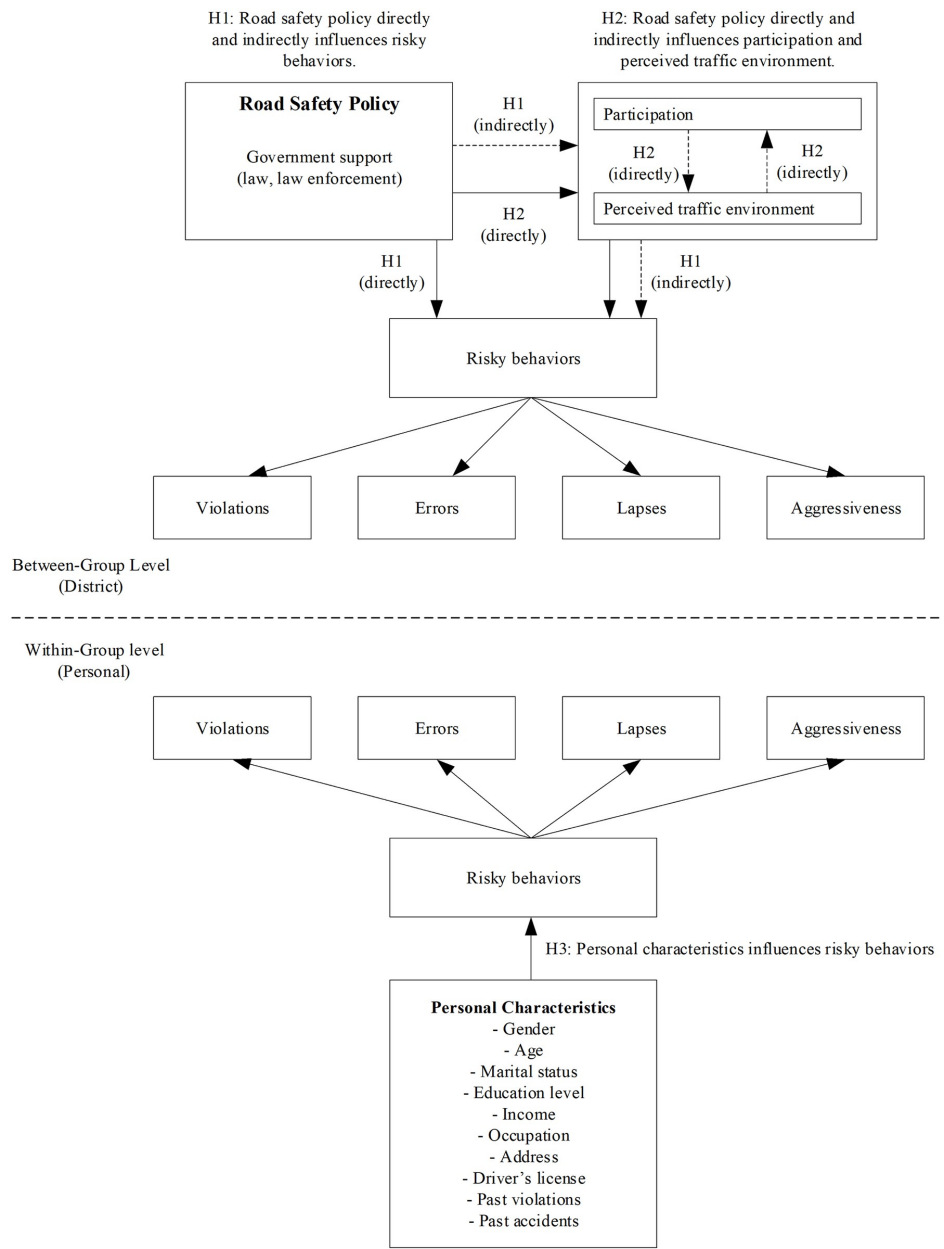
Analytical framework.

Road safety policy studies, which have a law, regulation, and enforcement, public sectors were developed and encouraged to solve the problem of unsafe driving. Population characteristics were considered as a different role and level compared to unsafe driving behavior, and road safety policy was part of overall behavior controlled management, while populations’ characteristics also influence behavior at the sub-level. Multilevel analysis is an interesting and alternative method over others, and is able to integrate both levels into the analysis. Therefore, the researchers designed the analytical framework (Fig 1), which indicated research questions and hypotheses for this study.

The research questions for this study were formulated as follows.

Can human factors be integrated into road safety policy in the form of multilevel analysis that affects the risky behaviors of Thai people? Which factors have direct influences? Which factors have significantly positive and negative influences?

Therefore, the analytical framework of this research allowed for the study of risky behaviors performed by drivers by grouping road safety policy and personal characteristics to analyze them together in a two-level analysis with the following hypotheses (Fig 1).

H1: Road safety policy directly and indirectly influences risky behaviors.
H2: Road safety policy directly and indirectly influences participation and perceived traffic environment.
H3: Personal characteristics influences risky behaviors.

The aims of this study are (1) to study and develop a multilevel analysis of risky behaviors (2), to investigate the influence of road safety policy on the risky behaviors of Thai people, and (3) to identify personal characteristics that affect to risky behaviors. The study’s results can be taken into consideration to support recent road safety policy, as well as various measures to let people in the area participate and improve the situation.

## 2. Materials and methods

### 2.1 Research procedures

**Fig 2:**
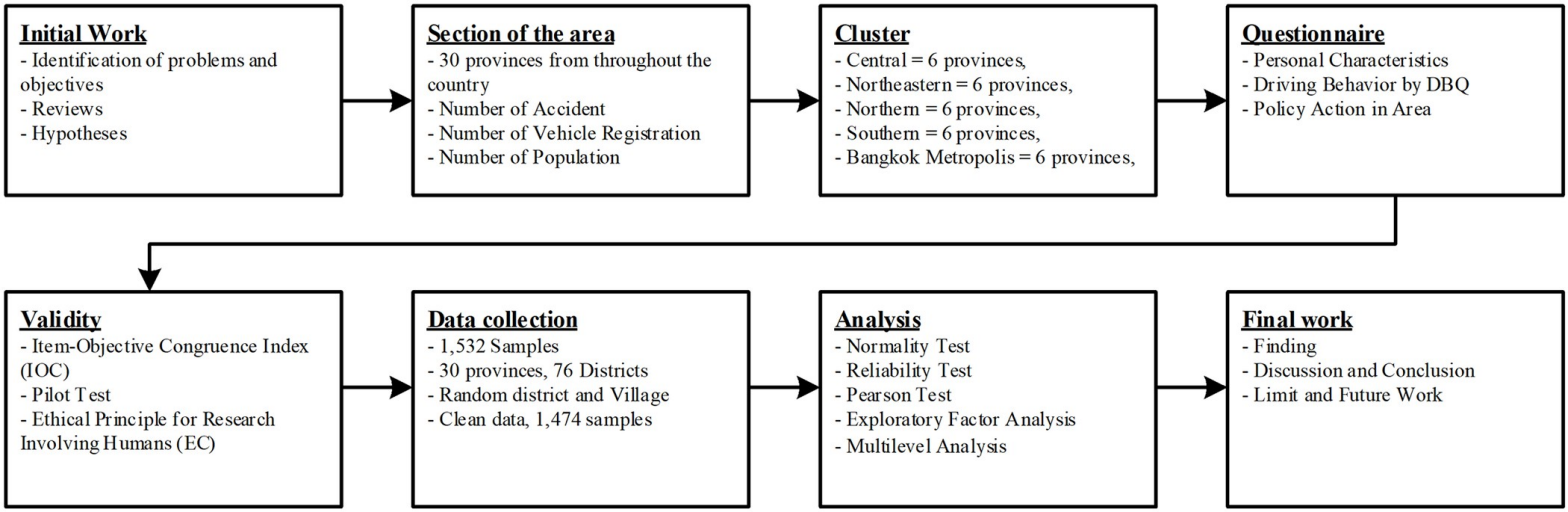
Research procedures.

Research procedures (Fig 2): The problem and objectives, as well as a literature review and the presentation of the hypotheses, were specified in our initial work. The data collection design was analyzed before developing the questionnaire, the accuracy of which was checked by experts. This phase consisted of a data collection trial, a request to conduct research on humans, data collection, a re-test for accuracy, and data analysis to answer the research questions and test the hypotheses. This paper ends with a presentation of the findings, as well as a discussion, conclusion, limitations, and directions for future work.

### 2.2 Materials and participants

#### 2.2.1 Sample cluster and data callection

**Fig 3:**
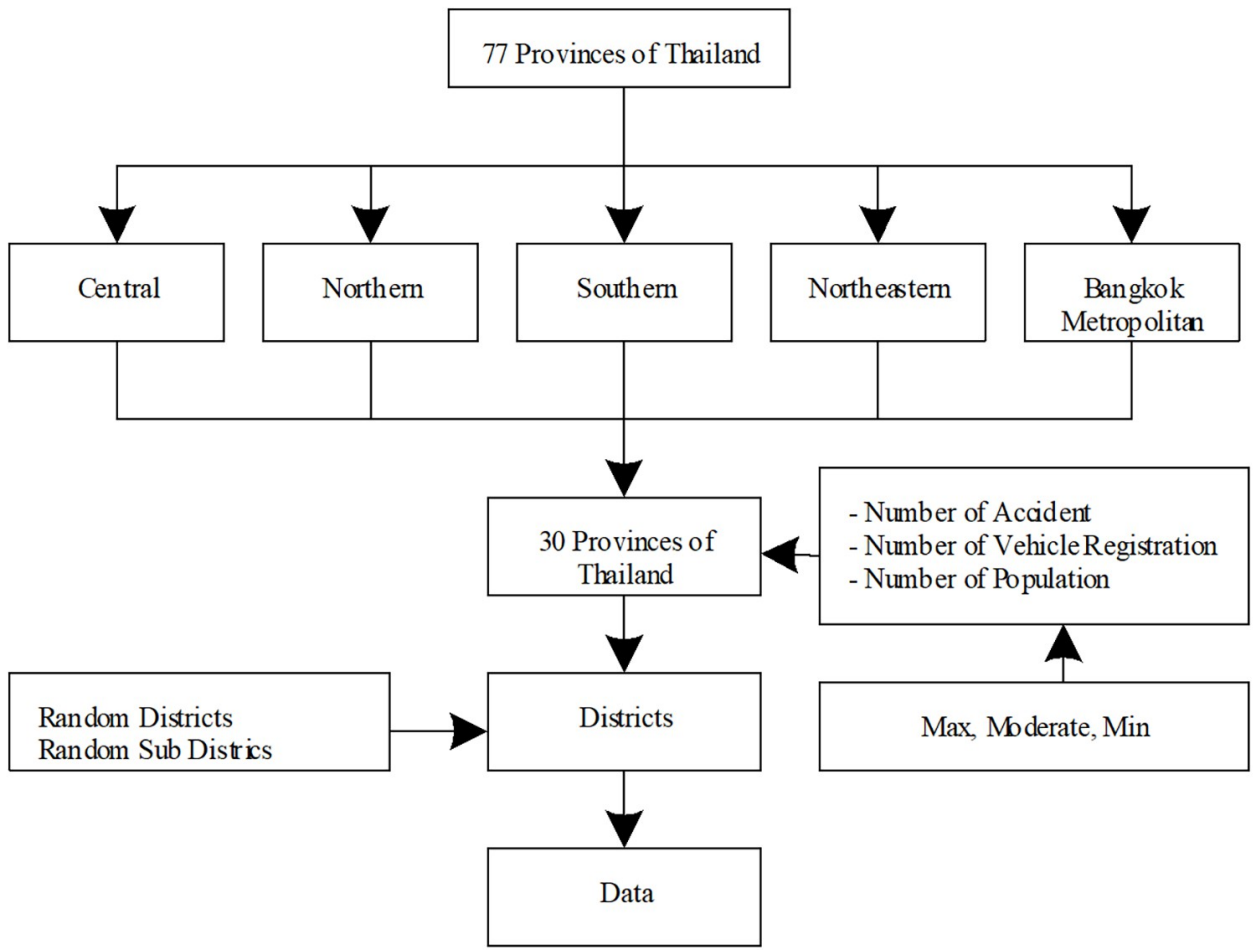
Data collection design.

The data collection area was selected to cover the required population for this study in 77 provinces in four regions, including the central, northeastern, northern, and southern regions. Even though the Bangkok Metropolitan Area was assigned to the central region, since it is the capital and an urban area with a dense population, we designated it as a separate study area. The researcher used specific criteria to select representative provinces in each region, consisting of statistics on the number of accidents, registered cars, and population size. Overall, 30 provinces were selected, with six of them in each region. Data were collected from both urban and rural areas, along with random data collected at the sub-district and village levels (Table 1 and Fig 3).

**Table 1:** Characteristics of the participants; *n* = 1,474.

Variable	Detail	Frequency	Valid Percent (%)
**Region**	Central: provinces by Chon Buri, Rayong, Nakhon Pathom, Saraburi, Kanchanaburi, and Prachin Buri.	• 6 provinces • 12 districts • 303 samples	• 20.0 • 15.8 • 20.6
	Northern: provinces by Chiang Mai, Chiang Rai, Nakhon Sawan, Phitsanulok, Phichit, and Phayao	• 6 provinces • 19 districts • 257 samples	• 20.0 • 25.0 • 17.4
	Southern: provinces by Nakhon Si Thammarat, Surat Thani, Phuket, Trang, Chumphon, and Phangnga	• 6 provinces • 12 districts • 300 samples	• 20.0 • 15.8 • 20.4
	Northeastern: provinces by Nakhon Ratchasima, Ubon Ratchathani, Surin, Kalasin, Nakhon Phanom, Mukdahan	• 6 provinces • 19 districts • 308 samples	• 20.0 • 24.0 • 20.9
	Bangkok Metropolitan Area: provinces by Bangkok, Samut Sakhon, Pathum Thani, Samut Prakan, Nonthaburi, and Samut Songkhram	• 6 provinces • 14 districts • 306 samples	• 20.0 • 18.4 • 20.8
**Gender**	Male	884	60.0
	Female	590	40.0
**Age**	• Average = 37.34 years • SD^a^ = 10.056 • Max = 78 • Min = 20		
**Family status**	Single	448	30.4
	Married	810	55.0
	Divorce	216	14.7
**Education**	Primary school	136	9.2
	Junior high school	198	13.4
	Senior high school	172	11.7
	Diploma	251	17.0
	Bachelor’s degree	641	43.5
	Master’s degree	63	4.3
	PhD	13	0.9
**Income**	• Average = 25,543.55 Baht/month • SD^a^ = 13,961.356 • Max = 200,000 • Min = 5,000		
**Occupation**	Student	71	4.8
	Civil servant/state-owned enterprise	85	5.8
	Private companies	603	40.
	Personal business/trading	406	27.5
	Farmer	109	7.4
	Contractor	165	11.2
	Housewife	27	1.8
	Other	8	0.5
**Address**	Urban	743	49.8
	Rural	740	50.2
**Driver’s license**	Yes	1,363	92.5
	No	111	7.5
**Violations within the past year**	No	422	28.6
	1–2 times	458	31.1
	3–4 times	269	18.2
	5–6 times	91	6.2
	> 6 times	234	15.9
**Past accidents within the past year**	No	1006	68.2
	once	375	25.4
	twice	81	5.5
	3 times	12	0.8

^a^standard deviation

After data collection area was considered; the sample size will be indicated for this study. Samples were chosen countrywide, including 1,532 sample sets. A total of 1,474 sample sets could be used for analysis (Table 2), consisting of the central (12 districts with 303 samples), northern (19 districts with 257 samples), southern (12 districts with 300 samples), and northeastern (14 districts with 308 samples) regions, as well as the Bangkok Metropolitan Area (14 districts with 306 samples), for a total of 76 districts with 1,474 samples.

**Table 2:** Personal characteristics for the within-group level model.

Variable	Personal dummy variable
**Gender**	Male (1), Female (0)
**Age**	< = 35 years old (1), > 35 years old (0)
**Family status**	Single (1), Other (0)
**Education**	< Bachelor (1), > = Bachelor (0)
**Income**	> 30,000 Baht (1), < = 30,000 Baht (0)
**Occupation**	Student (1), non-student (0)
**Address**	Urban (1), Rural (0)
**Driver’s license**	Have (1), Without (0)
**Past violations**	Have (1), Without (0)
**Past accidents**	Have (1), Without (0)

As for the characteristics of the participants (Table 1), 60% were male, their average age was 37.34 years old (SD = 10.056, max = 78, min = 20), and 55% were married. As for education level, 43.5% had a bachelor’s degree. The average income was around 25,543.55 Baht/month (SD = 13,661.356, max = 200,00, min = 5,000). Further, it private companies (40.9%) and personal business/trading (27.5%) were the majority group of the samples. The respondents lived in rural (50.2%) and urban (49.8%) areas at an equal ratio. Almost all respondents had a driver’s license (92.5%). In addition, the majority of respondents (68.2%) had no experiences with accidents.

For the analysis of SEM, Golob [53] suggested using proper numbers of a sample size in various ways, such as *n* = 200 [54, 55], *n* = 15, and *n* = 15 times the observable variable numbers [56], as well as *n* = 5 times the free parameters including the error term [57] and *n* = 10 times the free parameters [58]. Thus, when calculating from samples by region > 200, it would be proper to conduct the analysis as suggested earlier.

#### 2.2.2 Questionnaire

The DBQ [36] was used to gather data, covering four factors: violations, errors, lapses, and aggressiveness from Jomnonkwao et al. [39]. Such factors are unsafe driving behaviors. Data were collected using a 6-point Likert scale (0 = never and 5 = always) [59] to indicate the frequency of driving behaviors and to analyze the factor loading. If greater than 0.50, the indicator was considered for further study [36].

In addition, personal characteristics on the samples were collected, including gender, age, education level, career, income, residence, possession of a driver’s license, and driving experience (traffic law violations and accident record in the previous year). Data on accident prevention policies in the area consist of law enforcement, government support, the participation of people in the area, attitudes toward the place, and so forth.

Tool testing was conducted before actual data collection by using the Item-Objective Congruence Index (IOC) from seven professionals who could screen the research tools. Then, 100 sets of data were used for a trial of the data collection process. Data distribution testing was carried out by using a normal distribution [54], and the Cronbach’s alpha was checked to ensure that its values were higher than or equal to 0.7 [60]. This research was approved by the Ethics Committee for Research Involving Human Subjects, Pr: EC-63-52.

### 2.3 Multilevel analysis

Muthén [47] considered all levels of the variables studied to analyze them in one model consisting of two sub-models, including a between-group model, which has a causative relation to the macro variable, and a within-group model, which has a causative relation with the micro variable. Then, both sub-models were combined and examined in a multilevel model by creating a special latent variable as the average of the micro variable, since multilevel analysis has different variations of variables, which we wanted to study for both the between-group and within-group levels.

At the personal level (within-group), as seen in Table 2, the variables were gender, age, marital status, education level, income, occupation, address, driver’s license, past violations, and experiences with accidents. These sample characteristics are specified as dummy variables in the analysis. In addition, the between-group variable data are shown in Table 3. These are road safety policy (government support), participation, and perceived traffic environment.

**Table 3:** Sample statistics; *N* = 1,474.

Code	Variable	Mean	SD^a^	SK^b^	KU^c^
	**Road safety policy (government support)**				
GS1	There are constant and proper campaigns for the “Safe Drive, Maintain Traffic Discipline” program in your area.	4.83	0.869	0.140	−0.189
GS2	There is proper organizing of “Cooperative Operation Center of Road Accident Prevention and Reduction during the Festivals” in your area.	4.81	0.905	0.303	−0.521
GS3	There is organizing of “Community Barriers” by requesting participation from community volunteers (i.e., the Department of Local Administration, etc.) in your area.	4.94	0.981	−0.138	−0.254
GS4	There is a public relations effort for “Generous Drive, Maintain Traffic Discipline” via all channels in your area.	4.76	0.949	0.134	−0.210
GS5	There are public relations efforts and campaigns about replacing the use of personal vehicles with public buses in your area.	4.79	1.025	−0.092	0.085
	**Participation**				
PA1	There is the participation of people in the “Safe Drive, Maintain Traffic Discipline” program in your area.	4.66	1.059	0.111	−0.431
PA2	There is a constant operation by the government/private entities to implement “Safe Drive, Maintain Traffic Discipline” measures in your area.	4.55	1.105	0.019	−0.870
PA3	There is significant participation in the initiative to follow traffic laws along with traffic police during the festivals in your area.	4.61	1.155	−0.226	−0.669
PA4	There is proper speed management in communities/villages in your area.	4.58	1.236	−0.174	−0.676
PA5	There is 100% participation of wearing a helmet by government/school/private entities in your area.	4.56	1.254	−0.260	−0.718
PA6	There is constant control/inspection/deterrence (by the head of the sub-district, village, etc.) regarding drivers in your community.	4.42	1.270	−0.106	−0.920
	**Perceived traffic environment**				
PTE1	Road conditions in your area are convenient for driving and have always been improved.	2.58	1.112	0.705	0.871
PTE2	There are convenient traffic conditions for driving, as well as proper traffic management, in your area.	2.57	1.130	0.793	0.872
PTE3	There is environmental management along two-sided pavement for proper vision while driving in your area.	2.82	1.119	0.645	0.563
PTE4	There is a public transit system as a travel facility to decrease the use of personal vehicles in your area.	2.72	1.182	0.509	0.097
	**Driver Behavior Questionaire (DBQ)**				
V1	Turning left on a main road toward oncoming vehicles without reducing one’s speed or stopping one’s car at “STOP” signs.	1.77	0.710	0.590	−0.038
V2	Taking a chance and going through lights that have turned yellow before turning red.	1.89	0.787	0.465	−0.507
V3	Driving against the flow of traffic or going the wrong way on a one-way street.	1.81	0.813	0.490	−0.976
V4	Driving on the hard shoulder of roads.	2.02	0.858	0.081	−1.365
E1	Ignoring the “GIVE WAY” sign when driving on narrow roads and not letting a driver from the other lane proceed.	1.74	0.709	0.422	−0.949
E2	Not stopping the car at pedestrian crossings to allow people to cross a road.	1.81	0.786	0.463	−0.891
E3	Overtaking in a prohibited area, on a narrow road, or where signs prohibiting overtaking are present.	1.94	0.884	0.285	−1.301
L1	Forgetting the current gear that the car is in and checking it with the eyes or hands.	1.98	0.755	0.320	−0.436
L2	Dozing off while driving.	1.92	0.839	0.786	0.722
L3	Intending to turn on the widescreen wiper but turning on the light instead or vice versa.	2.05	0.918	0.340	−0.693
L4	Forgetting the car park position, such as at the department store.	2.20	1.010	0.556	−0.009
L5	Getting into the wrong lane when entering a roundabout or approaching a road junction.	1.99	0.975	0.875	0.606
G1	Getting involved in unofficial “races” with other drivers and not allowing other drivers to overtake you.	1.37	0.669	2.586	9.177
G2	Driving too close to the car in front of you and delivering flashing your lights to encourage the car in front to go faster or get out of the way.	1.45	0.666	1.564	2.577
G3	Becoming impatient or getting angry with slow drivers and needing to overtake them.	2.45	1.089	0.573	−0.052

^a^Standard deviation; ^b^Skewness; ^c^Kurtosis

## 3. Results

### 3.1 Descriptive statistics

The results used to check the basic statistical data from the 1,474 individuals in the sample are as follows. The road safety policy (government support) variable group’s mean values are between 4.76 and 4.94, the *SD* is between 0.869 and 1.025, the skewness is between −0.138 and 0.303, and the kurtosis is between −0.521 and 0.085. The participation variable group’s mean values are 4.42 and 4.66, the *SD* is between 1.059 and 1.270, the skewness is between −0.260 and 0.111, and the kurtosis is between −0.920 and −0.431. Moreover, the perceived traffic environment variable group’s mean values are 2.57 and 2.82, the *SD* is between 1.112 and 1.182, the skewness is between 0.509 and 0.793, and the kurtosis is between 0.097 and 0.872.

The risky behaviors variable’s mean values are between 1.37 and 2.45, the *SD* is between 0.666 and 1.089, the skewness is between 0.081 and 2.586, and the kurtosis is between −0.009 and 9.177. While considering skewness and kurtosis, which are used to indicate the sample distribution, we employed a maximum likelihood estimation. The most important condition is that the data had to have a normal distribution, using skewness and kurtosis, as well as normal distribution indicators. The skewness had to be less than 3.0, and kurtosis needed to be less than 10.0 [54], as presented in Table 3.

### 3.2 Exploratory factor analysis (EFA)

The EFA (Table 4) of the road safety policy, participation, and perceived traffic environment has a Kaiser–Meyer–Olkin (KMO) value of 0.864, which can be classified into three groups, including government support (GOV), consisting of variable codes GS1–GS5 with a Cronbach’s alpha of 0.932. Participation (PAR) is the factor consisting of variable codes PA1–PA6 with a Cronbach’s alpha of 0.960. In addition, there is the perceived traffic environment (PTE) factor consisting of variable codes PTE1–PTE4 with a Cronbach’s alpha of 0.927. The reliability value of the Cronbach’s alpha is more than 0.7 [60].

**Table 4:** Road safety policy results of EFA; KMO = 0.864.

Code	Component			Cronbach’s alpha
	1	2	3	
	(Government support: GOV)	(Participations: PAR)	(Perceived traffic environment: PTE)	
**GS1**	0.845			0.932
**GS2**	0.869			
**GS3**	0.876			
**GS4**	0.799			
**GS5**	0.662			
**PA1**		0.830		0.960
**PA2**		0.833		
**PA3**		0.837		
**PA4**		0.875		
**PA5**		0.893		
**PA6**		0.816		
**PTE1**			0.836	0.927
**PTE2**			0.831	
**PTE3**			0.937	
**PTE4**			0.902	

Extraction method: Principal component analysis

Rotation method: Varimax with Kaiser normalization

The EFA results of risky behaviors (Table 5) have a KMO value = 0.906, which can be classified into four groups, including violations (VIO), consisting of variable codes V1–V4 with a Cronbach’s alpha of 0.896. Errors consist of variable codes E1–E3 with a Cronbach’s alpha of 0.874. Lapses range from variable codes L1–L5 with a Cronbach’s alpha of 0.899. In addition, aggressiveness factors consist of variable codes G1–G3 with a Cronbach’s alpha of 0.761. The reliability of Cronbach’s alpha is more than 0.7 [60].

**Table 5:** Risky behavior results of EFA; KMO = 0.906.

	Component				
Code	1	2	3	4	Cronbach’s alpha
	Violations (VIO)	Errors (ERR)	Lapses (LAP)	Aggressiveness (AGG)	
V1	0.708				0.896
V2	0.981				
V3	0.537				
V4	0.527				
E1		0.880			0.874
E2		0.829			
E3		0.879			
L1			0.962		0.899
L2			0.730		
L3			0.569		
L4			0.659		
L5			0.686		
G1				0.795	0.761
G2				0.899	
G3				0.864	

Extraction Method: Principal component analysis

Rotation Method: Promax with Kaiser normalization

Table 6 shows the convergent validity test results by composite reliability (CR) and average variance extracted (AVE). The CR values for each factor range from 0.794 to 0.951, which is greater than 0.7 [61]. For the AVE values to be greater than 0.5 [61], all factor values must be between 0.744 and 0.846. The discriminant validity test result, which evaluated the relationship between inter-construct relations and the square root of AVE, was also considered. The square root of AVE, which is greater than the correlation values, is shown in bold on the diagonal of Table 6, and Fornell and Larcker [62] suggestions confirm this finding.

**Table 6:** Discriminnt validity, composite reliability (CR), and average variance extracted (AVE).

Factors	CR	AVE	Discriminnt validity						
			1	2	3	4	5	6	7
**1. GOV**	0.927	0.844	**0.919**						
**2. PAR**	0.951	0.836	0.669**	**0.914**					
**3. PTE**	0.906	0.833	0.309**	0.409**	**0.913**				
**4. VIO**	0.898	0.822	−0.238**	−0.438**	−0.020	**0.907**			
**5. ERR**	0.884	0.846	−0.154**	−0.425**	−0.086**	0.763**	**0.919**		
**6. LAP**	0.897	0.796	−0.074**	−0.314**	0.020	0.684**	0.671**	**0.892**	
**7. AGG**	0.794	0.744	−0.187**	−0.367**	−0.098**	0.567**	0.512**	0.430**	**0.863**

**. The correlation is significant at the 0.01 level (2-tailed)

Square root of average variance extracted shown on diagonal in bold

### 3.3 Multilevel analysis

A multilevel model with two levels of variables is used in the analysis to examine hypothetical models [47], including personal level and district level, the results are presented below.

#### 3.3.1 Intra-class correlations

The intra-class correlation (ICC) test is used to check data and also for multilevel analysis. Since the variables employed for multilevel analysis must vary at both the individual and organizational levels, they are appropriate for multilevel analysis. High ICC values mean that the correlation is also high. If the ICC values are less than 0.05, the personal characteristics have no variation at the organizational level. Therefore, such data do not need to be collected for multilevel analysis so that the ICC will be more than 0.05 [63].

In this study, Mplus 7.2 software [64] were analyzed ICC values, 15 observable variables including V1, V2, V3, V4, E1, E2, E3, L1, L2, L3, L4, L5, G1, G2, and G3; for ICC, the values are 0.213, 0.208, 0.176, 0.191, 0.194, 0.184, 0.214, 0.199, 0.192, 0.206, 0.214, 0.153, 0.395, 0.109, and 0.140, respectively. Such ICCs are high enough for multilevel analysis.

#### 3.3.2 Goodness of fit statistics

In this study, multilevel analysis for hypothesis testing (H1-H3) revealed that the model had goodness-of-fit statistics in terms of risky behaviors as follows: chi-square (χ^2^) = 79.201, degrees of freedom (df) = 40 causing the chi-square (χ^2^) /df = 1.98 < 3 [65], *p* value < 0.001, a root mean square of approximation (RMSEA) = 0.026 < 0.08 [66, 67], a comparative fit index (CFI) = 0.982 > 0.9 [65], a Tucker-Lewis index (TLI) value = 0.970 > 0.8 [68, 69], a standardized root mean residual (SRMR) between level = 0.028 and SRMR for within level = 0.018, at less than 0.08 for both levels [65].

#### 3.3.3 Between-group level (district-level)

**Fig 4:**
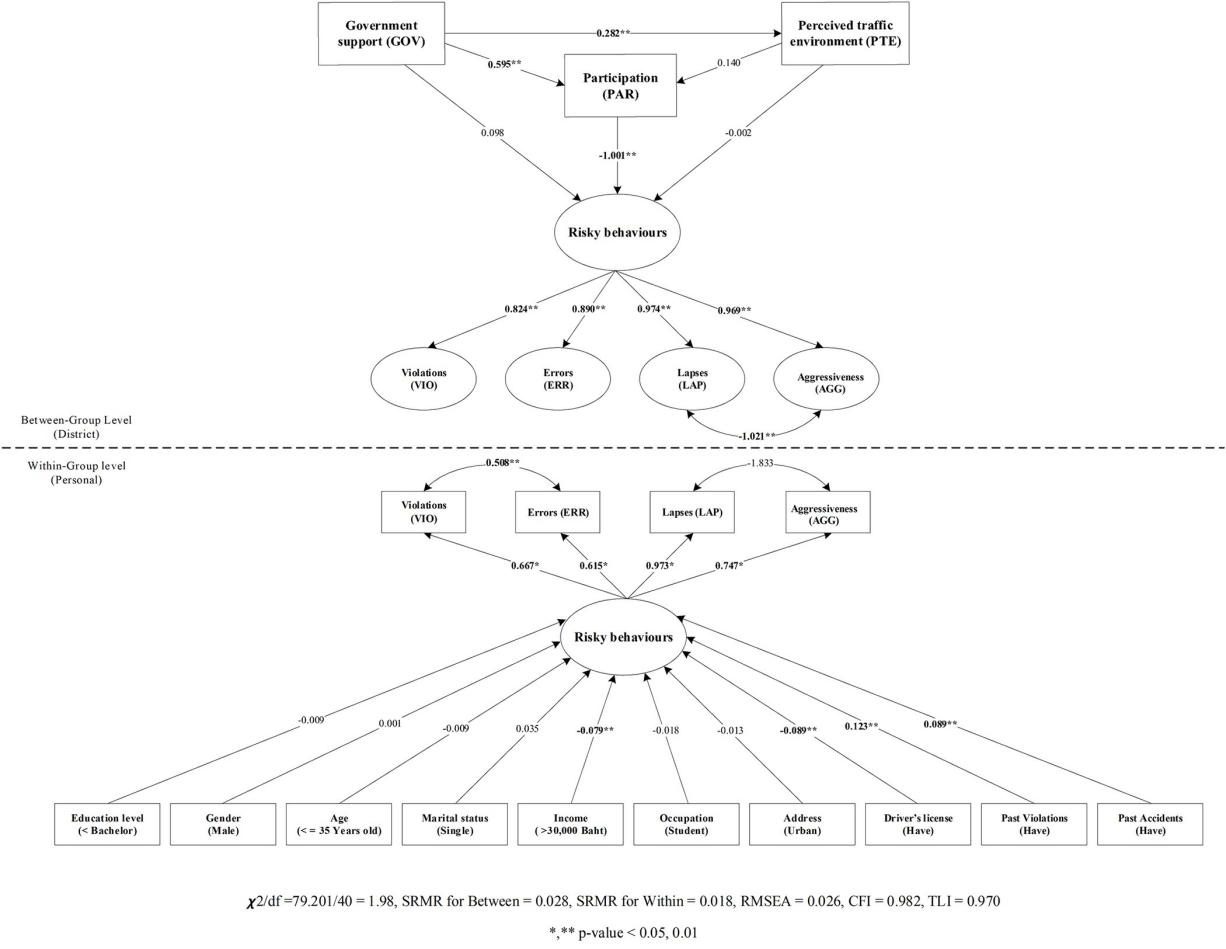
Risky behaviours modified model results.

Table 7 and Fig 4 explain the measurement model; while considering the importance of the factor of each observable variable in the district-level model or the between-group level model, we determined the factor loading of the observable variables using the risky behaviors measurement model; the errors valuable has the highest factor loading at 0.974, followed by violations (0.969), lapses (0.890), and aggressiveness (0.824).

**Table 7:** Model results for risky behaviors (unsafe actions).

	Between-group level (district-level)					Within-group level (personal-level)				
Variable	Est.	Est./S.E.	p value	RV	R^2^	Est.	Est./S.E.	p value	RV	R^2^
**Measurement model**										
**Risky behaviors were measured by:**										
**Violations (VIO)**	0.969	58.265	< 0.001**	0.061	0.939	0.667	9.568	< 0.001**	0.555	0.445
**Errors (ERR)**	0.974	53.124	< 0.001**	0.052	0.948	0.615	8.443	< 0.001**	0.622	0.378
**Lapses (LAP)**	0.890	20.927	< 0.001**	0.208	0.792	0.973	10.552	< 0.001**	0.054	0.946
**Aggressiveness (AGG)**	0.824	14.656	< 0.001**	0.321	0.679	0.747	8.965	< 0.001**	0.442	0.558
**Path model**										
**H1: Road safety policy directly and indirectly influences risky behaviors.**										
**Government support (GOV) → risky behaviors**	0.098	1.049	0.294							
**Participation (PAR) → risky behaviors**	−1.001	−17.389	< 0.001**							
**Perceived traffic environment (PTE) → risky behaviors**	−0.002	−0.026	0.979							
**H2: Road safety policy directly and indirectly influences Participation and perceived traffic environment.**										
**Government support (GOV) → participation (PAR)**	0.595	5.758	< 0.001**							
**Government support (GOV) → perceived traffic environment (PTE)**	0.282	2.675	0.007**							
**Perceived traffic environment (PTE) → participation (PAR)**	0.140	1.233	0.217							
**H3: Personal characteristics influences risky behaviors.**										
**Gender → risky behaviors**						−0.009	−0.413	0.679		
**Age → risky behaviors**						0.001	0.036	0.971		
**Marital status → risky behaviors**						−0.009	−0.301	0.764		
**Education level → risky behaviors**						0.035	1.024	0.306		
**Income → risky behaviors**						−0.079	−3.671	< 0.001**		
**Occupation → risky behaviors**						−0.018	−0.744	0.457		
**Address → risky behaviors**						−0.013	−0.400	0.689		
**Driver’s license → risky behaviors**						−0.089	−2.943	0.003**		
**Past violations → risky behaviors**						0.123	3.431	0.001**		
**Past accidents → risky behaviors**						0.089	3.539	0.001**		

Model fit statistics: χ^2^ = 79.201; df = 40; *p* < 0.001; RMSEA = 0.026; CFI = 0.982; TLI = 0.970; SRMR_Within_ = 0.018; SRMR_Between_ = 0.028.

** Denotes significance at the 0.01 level and supports the hypotheses

Path model; the result is considered bases on the hypotheses. While considering the influence scale of the predicted variables at the district level, which affected risky behaviors for H1 and H2 testing, we found that participation directly affected risky behaviors on a statistically significant level of 0.01, with an influence scale with a negative coefficient value of −1.001 (*p* value < 0.001). Government support directly impacted risky behaviors, with an influence scale of a positive coefficient value of 0.595 (*p* value < 0.001). Thus, government support also indirectly affected percived traffic environment, with an influence scale of a positive coefficient value of 0.282 (*p* value < 0.001).

#### 3.3.4 Within-group level (personal-level)

Mesurement model; the result of within-group level or personal-level, risky behaviors measurement model while considering the importance of the factor of each observable variable, we observed that the factor loading of the observable variables for risky behaviors is highest in lapses (0.973), followed by aggressiveness (0.747), violations (0.667), and errors (0.615).

Path model; while considering the influence scale of the predicted variable at the personal-level for H3, which affected risky behaviors, we discovered that income (> 30,000 baht) influenced the scale with a negative coefficient value of −0.079 (*p* value < 0.001) and having a driver’s license affected the scale with a negative coefficient value of −0.089 (*p* value = 0.003). Past violations and past accidents resulted in risky behaviors and had an influence scale with positive coefficient values of 0.123 (*p* value = 0.001) and 0.089 (*p* value = 0.001), respectively, as presented in Table 7 and Fig 4.

## 4. Discussion

Human factors are the main causes of accidents [7] and are relevant to unsafe actions (risky behaviors) and safety violations [33]. The DBQ [36] was used to gather data to assess risky behaviors performed by Thai people while driving. We also considered the road safety policy, which is specified and enforced countrywide to reduce accidents, as well as to create and support safe driving. In reference to the results regarding unsafe actions performed by Thai people, we measured this aspect using four factors (violations, errors, lapses, and aggressiveness) [14, 16, 17, 35]. In contrast, violations can be measured by four indicators of driving behavior, errors can be assessed by three indicators, lapses can be evaluated by five indicators, and aggressiveness can be measured by three indicators of the DBQ, which consists of a total of 43 questions [36].

A multilevel model with two levels of variables district-level and personal-level is used for analysis to evaluate the research hypothesis.

District-level; the results from the co-integration of the road safety policy revealed that government support and perceived traffic environment had no direct influence on risky behaviors, which is consistent with the work Stanojević [4], Weiss and Freels [6], who found that traffic enforcement has no influence on accidents and driving behavior. The finding of road safety policies might not be consistent with previous studies, which indicate a decrease in unsafe behaviors. In contrast, road safety policy in this study had an indirect effect by passing through populations’ cooperation [9], which an important strategy that led to the achievement of road safety policies [10].

Participation had a significantly negative influence at a 99% reliability level. While considering indirect influences, we found that government support had a significantly positive influence on participation and percived traffic envioronment. These are government support, safety policy distribution, the organization of community barriers for local leaders, and the presence of authorized people who effectively enforce the law, which affect the participation of people in the area, as indicated by Yannis et al. [5] and Landge et al. [9], who determined that law enforcement led to a decrease in accidents. Stanojević et al. [4] also mentioned that if law enforcement and support of safety are negligent, it will increase risky behaviors or unsafe behavior.

Personal-level; the result found that an income > 30,000 Baht led to a significant decrease in risky behaviors at a 99% reliability level. Regarding having a driver’s license, we discovered that people who had driver’s licenses had lower levels of risky behaviors at a 99% reliability level, a finding in line with previous studies, such as that of Ulmer et al. [70], who studied a group of new drivers. The findings indicated that receiving training before obtaining a driver’s license led to a 9% decrease in vehicle accidents. Mayhew et al. [71] noted that training and testing processes are effective methods to ensure better driving. Shope and Molnar [72] found that, after a follow-up study after four years, there was a significant decline in the risk and occurrence of accidents (65% reliability level). Fohr et al. [44] asserted that having a driver’s license resulted in a decrease in the accident rate of the general population, especially among 15- and 16-year-old teens due to decreased risk, thus lowering the number of accidents. Moreover, Braitman et al. [73] discovered that such factors helped to reduce the risk of accidents.

However, the results of testing and training processes before obtaining a driver’s license differ across countries. If samples of such training and testing programs from successful countries are observed to identify their positive features and to integrate them properly in Thailand, this will support the road safety policy more solidly, particularly for violations and errors [74].

While considering past violations, we discovered that a group of people who violated traffic laws in the past year caused an increase in unsafe actions with 99% reliability, in line with the work of Stanojević [37] et al., Parker et al. [75]. In addition, while considering past accidents, we found that a group of people who experienced an accident in the past year performed more risky behaviors at a 95%–99% reliability level. However, age, gender, family status, education level, occupation, and address had no influence.

The limitation of this study is that the research method is based on self-reported measures for all variables. It brings up the issue of common method bias (CMB), which is prejudice brought using of measuring techniques from informants, time, place, and measurement feature (using a Likert scale). CMB generates measurement inaccuracies in indicators referred to as common method variance (CMV), which can influence analytical results [76]. Furthermore, distribution of samples in research studies do not cover teenagers, who are under 20 years of age, even though they can drive. As the questionnaire required permission from their parents, access to data from this group was restricted.

## 5. Conclusion

This study suggests that, at the district level or the between-group level, the participation factor directly resulted in a significant decrease in risky behaviors, whereas government support indirectly affected risky behaviors through participation. Thus, the increase in safety policy support in the area played a role in encouraging public participation, leading to a significant decrease in unsafe driving behaviors as well.

At the personal-level or the within-group level, we found that income and the possession of a driver’s license triggered a significant decrease in risky behaviors, especially among groups of people with driver’s licenses. Conversely, past violations and accidents directly led to a significant rise in risky behaviors, particularly among groups of people who had violated traffic laws. This research indicates that understanding and solving unsafe driving at the spatial level can be achieved with the support of different measures to help people in the area cooperate and improve the situation. Moreover, we should take into account people with driver’s licenses by offering useful training and supporting them to decrease traffic law violations, as well as informing them about accidents. These steps would help decrease unsafe driving behaviors.

The results suggest that public participation played a role in the significant decrease in unsafe actions (risky behaviors). Meanwhile, even though government support has no direct influence on outcomes, it indirectly and significantly affects people. For future research, we recommend studying supporting guidelines from the government sector to identify the most effective policies and which ones can be measured for outcomes, thus promoting security and consistency for people participating in each area, as well as helping governments to identify which areas are appropriate role models for safety-related policymaking.

## Supporting information

S1 Dataset (XLSX)
